# α-Actinin-4-Mediated FSGS: An Inherited Kidney Disease Caused by an Aggregated and Rapidly Degraded Cytoskeletal Protein

**DOI:** 10.1371/journal.pbio.0020167

**Published:** 2004-06-15

**Authors:** June Yao, Tu Cam Le, Claudine H Kos, Joel M Henderson, Phillip G Allen, Bradley M Denker, Martin R Pollak

**Affiliations:** **1**Renal Division, Department of Medicine, Brigham and Women's Hospital and Harvard Medical SchoolBoston, MassachusettsUnited States of America; **2**Department of Pathology, Brigham and Women's Hospital and Harvard Medical SchoolBoston, MassachusettsUnited States of America; **3**Hematology Division, Department of Medicine, Brigham and Women's Hospital and Harvard Medical SchoolBoston, MassachusettsUnited States of America

## Abstract

Focal segmental glomerulosclerosis (FSGS) is a common pattern of renal injury, seen as both a primary disorder and as a consequence of underlying insults such as diabetes, HIV infection, and hypertension. Point mutations in theα-actinin-4 gene *ACTN4* cause an autosomal dominant form of human FSGS. We characterized the biological effect of these mutations by biochemical assays, cell-based studies, and the development of a new mouse model. We found that a fraction of the mutant protein forms large aggregates with a high sedimentation coefficient. Localization of mutant α-actinin-4 in transfected and injected cells, as well as in situ glomeruli, showed aggregates of the mutant protein. Video microscopy showed the mutant α-actinin-4 to be markedly less dynamic than the wild-type protein. We developed a “knockin” mouse model by replacing *Actn4* with a copy of the gene bearing an FSGS-associated point mutation. We used cells from these mice to show increased degradation of mutant α-actinin-4, mediated, at least in part, by the ubiquitin–proteasome pathway. We correlate these findings with studies of α-actinin-4 expression in human samples. “Knockin” mice with a disease-associated *Actn4* mutation develop a phenotype similar to that observed in humans. Comparison of the phenotype in wild-type, heterozygous, and homozygous *Actn4* “knockin” and “knockout” mice, together with our in vitro data, suggests that the phenotypes in mice and humans involve both gain-of-function and loss-of-function mechanisms.

## Abstract 2

Transgenic experiments in mice suggest that the human kidney disorder, focal segmental glomerulosclerosis (FSGS), is a result of both gain- and loss-of-function mechanisms

## Introduction

In humans, *ACTN4* mutations cause a form of focal segmental glomerulosclerosis (FSGS) (Kaplan et al. 2000). This lesion, which describes a pattern of injury characterized by regions of sclerosis in some renal glomeruli, is a common finding in kidney disease from a wide range of primary disorders, including HIV infection, diabetes, and hypertension (Ichikawa and Fogo 1996; Somlo and Mundel 2000).

The four α-actinin genes encode highly homologous proteins that normally form approximately 100 kDa head-to-tail homodimers. While the best-defined function of α-actinin-4 is to cross-link and bundle actin filaments, α-actinins have been found to interact with a large and diverse set of other proteins (Honda et al. 1998; Takada and Beggs 2002). α-Actinin-2 and α-actinin-3 are located predominantly in the sarcomere, while α-actinin-1 and α-actinin-4 are widely expressed. In the human kidney, only α-actinin-4 expression is detected (Kaplan et al. 2000).

Human *ACTN4*-associated FSGS is inherited in an autosomal dominant pattern. By contrast, mice homozoygous for *Actn4* null alleles have glomerular disease, while heterozygous *Actn4* null mice have no readily apparent phenotype (Kos et al. 2003). Transgenic mice harboring a mutant *Actn4* targeted to the glomerular podocyte have an FSGS-like lesion, although it is not clear whether this is due to the dominant effect of the mutant *Actn4* per se or to dysregulated *Actn4* expression in this cell (Michaud et al. 2003).

We previously showed that FSGS-associated *ACTN4* mutations increase the binding of α-actinin-4 to F-actin (Kaplan et al. 2000). This was confirmed independently by different methodology (Michaud et al. 2003). However, the relationship between altered actin binding and disease is unclear. Of particular interest is whether the human disease is due to a gain-of-function effect of mutations on protein function or due to a partial loss of normal function. We therefore performed a series of experiments to help us understand the biological consequences of mutations in *ACTN4*. We demonstrate here that mutant α-actinins exhibit altered structural characteristics, localize abnormally, and have significantly diminished half-life. By developing a mouse model harboring a disease-associated point mutation, we confirm the pathologic effect of this mutation on glomerular function. Our results suggest that the major effects of *Actn4* mutations are protein misfolding and accelerated degradation, leading to loss of normal α-actinin-4 function, α-actinin-4 aggregation, and progressive kidney disease.

## Results

### Mutant α-Actinin-4 Conformation

**Figure 1:**
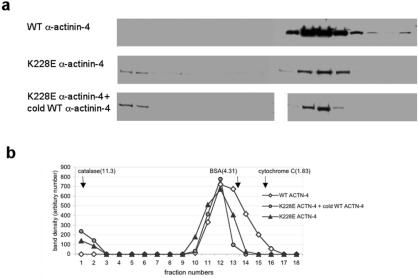
Mutant α-Actinin-4 Sediments Abnormally (A) Pattern of sedimentation of α-actinin-4 in a 10%–40% sucrose gradient. Results shown are for wild-type α-actinin-4, K228E α-actinin-4, and K228E α-actinin-4 after addition of excess cold (wild-type) α-actinin. A fraction of the mutant α-actinin-4 sedimented at least as quickly than the highest molecular weight marker, catalase, which has a sedimentation coefficient of 11.3. This was observed with all of the other mutants tested as well (data not shown), but never with labeled wild-type α-actinin-4. Addition of cold α-actinin did not alter the sedimentation pattern seen with the mutant form of α-actinin-4. (B) Results illustrated graphically. Units are arbitrary.

In overlay assays (performed as per Chan et al. 1998; data not shown), mutant α-actinin-4 is able to bind both mutant and wild-type α-actinin-4. Since mutant α-actinin-4 polypeptides are able to interact, we used sucrose gradient assays to examine whether the mutant α-actinin-4 dimerizes normally. We produced radiolabeled in vitro translated wild-type and mutant α-actinin-4 (K228E, T232I, R235P) and subjected these proteins to centrifugation in 5%–20% sucrose gradients. We found that all of the wild-type α-actinin-4 eluted as a single peak, as expected. By contrast, approximately 80% mutant α-actinin-4 eluted at the same peak as in wild-type, while about 20% of the mutant protein eluted much more quickly and peaked in the first fraction. We then used a 10%–40% sucrose gradient in an attempt to better characterize this small peak. Again, in multiple experiments, about 20% of the mutant α-actinins eluted in the first fractions. α-Actinin has a sedimentation coefficient of 6.4 (Feramisco and Burridge 1980), and both wild-type and the majority (approximately 80%) of mutant actinins migrated in this position. However, a significant fraction of mutant α-actinin-4 had a sedimentation coefficient equal to or greater than 11.3, the sedimentation coefficient of catalase (Figure 1). This extremely high sedimentation rate suggests either the presence of large α-actinin-4 multimers or the existence of large (and perhaps insoluble) aggregates. When these experiments were performed in the presence of a large excess of cold α-actinin, this rapidly sedimenting fraction of mutant α-actinin was unchanged, showing that the abnormally conformed mutant α-actinin-4 could not be shifted back into normally sedimenting mutant/wild-type heterodimers (Figure 1).

### α-Actinin-4 Behavior in Cells

**Figure 2:**
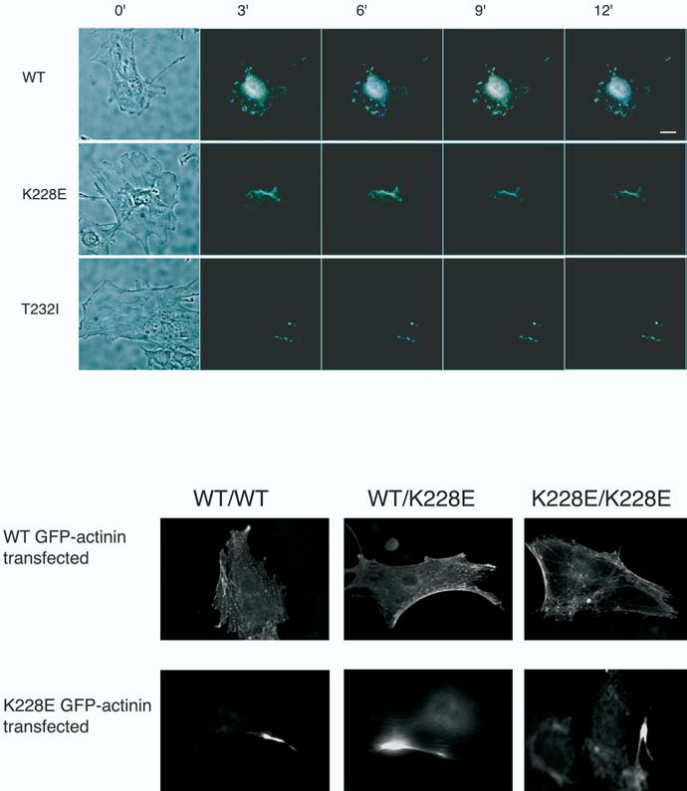
Mutant α-Actinin-4 Behavior in Cells (A) Mutant and wild-type α-actinin-4 show different localization and dynamics when expressed in a conditionally immortalized differentiated mouse podocyte cell line. Differentiated podocytes were injected in the nucleus with equal concentrations of expression plasmid for GFP fusions of mutant and wild-type actinins. At 2–4 h after injections, cells were imaged and both phase and fluorescence images recorded as described in the Materials and Methods. To illustrate changes in distribution of the fluorescence signal, three fluorescence images each 1 min apart were overlaid as red, green, and blue panes. Areas of fluorescence that were the same in all panes show as white, while dynamic areas are indicated by the color. The top panel indicates the initial phase and overlain dynamic fluorescence images of wild-type α-actinin-4, while the bottom two panels illustrate characteristic results for mutants K228E and T232I at 3 min time intervals. (See Videos S1–S3.) (B) Transfections in podocytes derived from mutant and wild-type mice. When transfected into conditionally immortalized podocytes of all three α-actinin-4 genotypes (+/+, K228E/+, or K228E/K228E), wild-type GFP–α-actinin-4 shows diffuse cytoskeletal localization. Mutant GFP–α-actinin-4 shows a similar alteration in localization when expressed in these three cells types.

We performed both transfection and nuclear injection studies in a conditionally immortalized podocyte cell line to look at the effect of disease-associated mutations on α-actinin-4 localization. Irrespective of the method used to express the mutant α-actinins in cells, we found altered localization of the mutants. Consistent with the altered sedimentation observed in vitro, mutant α-actinin-4 formed localized aggregates when expressed in cells. We used video microscopy to view the fate of the green fluorescent protein (GFP)–α-actinin-4 after nuclear injection of the cDNA. Similar results were observed in three independent sets of experiments. In each experiment, 15–35 cells were microinjected in the nucleus with plasmid DNA; of these, five to ten cells showed signal at 4–6 h after injection. Consistently, the mutants behaved abnormally, were unevenly distributed in the cell cytoplasm, and were much less dynamic compared with the wild-type proteins (Figure 2A; see also Videos S1–S3). These findings were consistent with the indirect immunofluorescence (IF) studies performed in *Actn4* ^K228/K228^ mice (see below).

We transfected conditionally immortalized podocytes derived from *Actn4* ^+/+^, *Actn4* ^K228E/+^, and *Actn4* ^K228E/K228E^ mice with either wild-type GFP–α-actinin-4 or K228E mutant GFP–α-actinin-4 (Figure 2B). The wild-type GFP–α-actinin-4 showed diffuse cytoplasmic localization in podocytes of all three genotypes. By contrast, mutant α-actinin-4 showed a similar aggregated appearance in all three cell types. This suggests the absence of a strong dominant effect of the mutant protein on the wild-type, as the endogenous K228E actinin does not alter the localization of the wild-type GFP-tagged protein.

**Figure 3:**
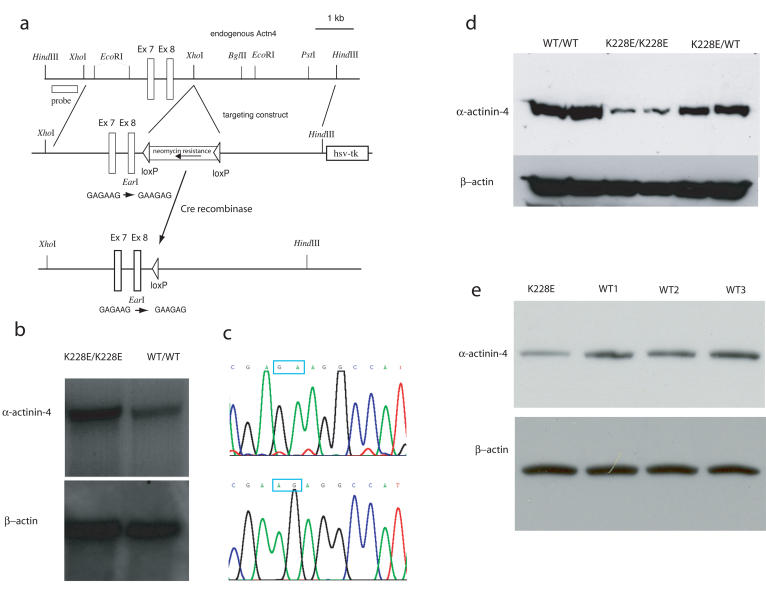
“Knockin” Mouse Model (A) Targeting construct. As we have described elsewhere (Kos et al. 2003), targeting initially resulted in a “knockout” allele, due to disruption of normal transcription, presumably by the intronic *loxP*-flanked neomycin resistance cassette. After breeding to *Cre* transgenic mice, this neomycin cassette was excised, as illustrated. (B) Northern blot analysis using kidney total RNA illustrates that the expression of the *Actn4* transcript in K228E/K228E is similar to the expression in wild-type mice. (C) RT-PCR and sequencing of the relevant portion of *Actn4* exon 8 confirms that the transcript in mice homozygous for the targeted allele contains the desired point mutation (top, wild-type; bottom, targeted). (D) Western blot showing markedly decreased expression of α-actinin-4 protein in K228E/K228E homozygous mice and moderately decreased expression in heterozygotes. Shown are blots using protein from cultured fibroblasts. Results were similar using protein extracted from lung, brain, liver, and kidney (data not shown). β-actin control is shown below. (E) Western blot showing expression of α-actinin-4 in lymphocytes from a human K228E/+ heterozygote (Kaplan et al. 2000) and three wild-type controls (two related, one unrelated). β-actin control is shown below.

We developed α-actinin-4 “knockin” mice using the methods of homologous recombination in embryonic stem cells. Previously we developed an *Actn4* “knockout” mouse (Kos et al. 2003). As indicated schematically in Figure 3A, mating these mice with germline *Cre* transgenic mice produced offspring in which an intronic *loxP*-flanked neomycin resistance cassette had been excised. We bred these heterozygous mice (*Actn4* ^K228E/+^) to generate litters with wild-type, heterozygous, and *Actn4* ^K228E/K228E^ mice. We genotyped mice by testing for the presence or absence of an engineered silent EarI site as described previously (Kos et al. 2003). The homozygous K228E mice, in contrast to the *Actn4*-deficient model, demonstrated normal levels of *Actn4* mRNA expression (Figure 3B). RT-PCR and sequencing of the transcript confirmed that the K228E allele was in fact expressed in the homozoygous mice. However, in multiple tissues tested (kidney, lung, leukocytes, brain), as well as in fibroblasts derived from these mice, α-actinin-4 protein expression was markedly reduced, with an approximately 90% reduction in protein expression in *Actn4* ^K228E/K228E^ homozygotes and an approximately 50% reduction in *Actn4* ^K228E/+^ heterozygotes (Figure 3D). Lymphocytes from a human subject heterozygous for one *ACTN4* K2228E mutation similarly had an approximately 50% reduction in the amount of α-actinin-4 compared with related and unrelated controls (Figure 3E).

### α-Actinin-4 Degradation

**Figure 4:**
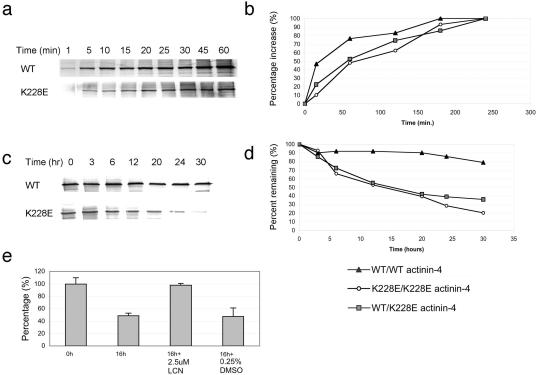
α-Actinin-4 Synthesis and Degradation (A and B) Synthesis of α-actinin-4 by wild-type and K228E/K228E fibroblasts. The rate of increase in the accumulation of mutant and wild-type α-actinin-4 is similar, as indicated by the superimposed shapes of the synthesis curves. (C and D) Pulse–chase experiments showing degradation of α-actinin-4 in K228E/K228E cells. Half-life of wild-type α-actinin-4 is greater than 30 h. Half-life of mutant α-actinin-4 is approximately 15 h. (E) Half-life of K228E mutant α-actinin-4 is restored to near-normal levels by the addition of lactacystin. Shown is labeled α-actinin-4 levels, expressed as a percentage of α-actinin-4 at time 0 and at 16 h and in the presence of 2.5 μM lactacystin in DMSO or in DMSO alone.

We observed decreased mutant α-actinin expression in an immortalized knockin mouse fibroblast homozygous for the *Actn4* K228E mutation, compared with the wild-type fibroblast (Figure 3D). To help determine the fate of the mutant α-actinin, we performed pulse and pulse–chase studies. We labeled *Actn4* ^K228E/K228E^ fibroblasts, *Actn4* ^K228E/+^ fibroblasts, and wild-type fibroblasts with [^35^S]methionine for different timepoints (pulse) following incubation in methionine-deficient medium. In order to trace the newly synthesized ^35^S-labeled α-actinins, we used an α-actinin-4-specific antibody to immunoprecipitate α-actinin-4 from the cell lysates. (We detected no α-actinin-4 in the cell pellets.) As shown in Figure 4A, we found that at any timepoint, there was less mutant than wild-type α-actinin-4 synthesized. However, as shown in Figure 4B, the rates of increase in labeled α-actinin-4 were similar, suggesting that the low expression level of mutant α-actinin-4 is not due to a defect in synthesis. We then labeled the mutant and wild-type fibroblasts with [^35^S]methionine for 3 hours (pulse), following incubation in methionine-deficient media, and then incubated the cells in media containing excess cold methionine (chase) for different timepoints to follow the degradation of the newly synthesized α-actinin-4. As shown in Figure 4C and 4D, mutant α-actinin-4 degraded at a much faster rate than did the wild-type protein. The estimated half-life of mutant α-actinin-4 is about 15 h, while the half-life of the wild-type α-actinin-4 is much greater than 30 h. Three replicate experiments gave the same results. The rapid degradation of the K228E mutant α-actinin-4 is reversed by treatment with lactacystin (Figure 4E), indicating that this mutant form of α-actinin-4 is degraded through the ubiquitin–proteasome pathway.

### In Vivo Phenotype

**Figure 5:**
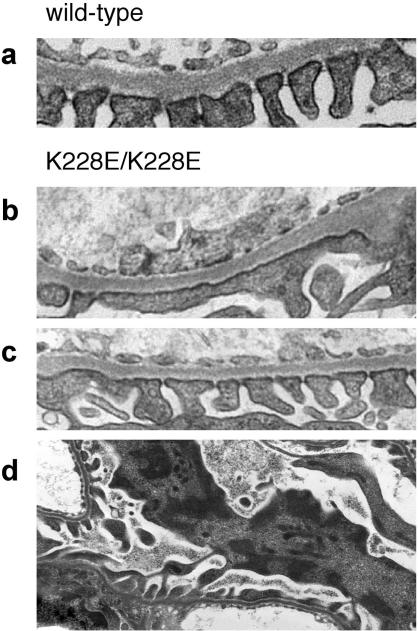
In Vivo Phenotype Electron micrographs from *Actn4* wild-type (A) and *Actn4* ^K228E/K228E^ mice (B–D). As shown, *Actn4* ^K228E/K228E^ mice were found to have abnormalities that were typically focal, with some areas of podocyte foot process effacement (B), as well as areas that appeared essentially normal (C). Bottom image ([D] using tannic acid counterstaining) illustrates electron-dense deposits observed in several podocyte cell bodies in *Actn4* ^K228E/K228E^ mice. No such deposits were observed in wild-type or heterozygous mice.

We performed standard histologic analyses of kidneys from *Actn4* ^K228/K228E^ mice, as well as *Actn4* ^K228/+^ and wild-type littermates. In *Actn4* ^K228/K228E^ mice as old as 13 mo, we saw no abnormalities by light microscopy with periodic acid–Schiff (PAS) and hematoxylin-and-eosin (H & E) staining. All of the 11 *Actn4* ^K228/K228E^ kidneys examined by electron microscopy had abnormalities in podocyte structure. Typically, these consisted of focal areas of foot process effacement (Figure 5A). By contrast, we found mild abnormalities in one of 13 wild-type and one of nine *Actn4* ^K228E/+^ littermates. Mice were typically genotyped at or shortly before the time of weaning (at approximately 3 wk of age). Only 10% (23 of 231) of the offspring of crosses between heterozygous mice were homozygous for the K228E change, suggesting increased peri- or neonatal lethality in the homozygous mice, similar to what we have observed in *Actn4* null mice (Kos et al. 2003). In *Actn4* ^K228/K228E^ mice, we frequently observed the appearance of abnormal electron-dense structures in the podocyte cell bodies (Figure 5D).

**Figure 6:**
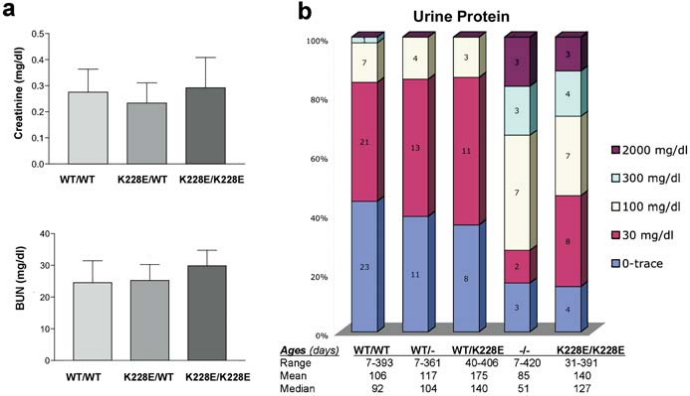
Biochemical Characteristics of Mutant Mice (A) Average BUN and creatinine levels in *Actn4* ^K228E/–^ (*n* = 12), *Actn4* ^+/+^ (*n* = 8), and *Actn4* ^K228E/K228E^ (*n* = 12) mice at the time of sacrifice. Differences were not statistically significant. Error bars show standard deviation. (B) Summary of proteinuria in wild-type, *Actn4* ^+/–^, *Actn4* ^K228E/–^, *Actn4* ^–/–^, and *Actn4* ^K228E/K228E^ mice, measured by albumin dipstick. Distribution of measurements are illustrated graphically for each genotype.

We measured urine protein excretion in mice of five different genotypes: wild-type (*Actn4* ^+/+^), heterozyogtes for either a null or K228E allele (*Actn4* ^+/–^ and *Actn4* ^K228E/+^), and homozoygotes for either a null or K228E allele (*Actn4* ^–/–^ and *Actn4* ^K228E/K228E^). Results were quite variable within each genotypic group of mice (likely reflecting differences in age and genetic background). However, the overall pattern of protein excretion was similar in the *Actn4* ^+/+^, *Actn4* ^+/–^, and *Actn4* ^K228E/+^ mice, while both groups of homozygous mutant mice (*Actn4* ^–/–^ and *Actn4* ^K228E/K228E^) had significantly greater—and similar—degrees of proteinuria (Figure 6B). We did not see significant differences in serum creatinine levels or blood urea nitrogen levels (BUN) between mice of the three genotypes (Figure 6A), nor did we identify any single mutant mouse with significant BUN or creatinine elevations.

To determine whether the K228E point mutation altered α-actinin-4 localization in vivo, we performed indirect IF studies. As shown in Figure 6A, α-actinin-4 appears to be mislocalized and aggregated in *Actn4* ^K228E/K228E^ kidneys. By contrast, we see no difference in the expression of slit-diaphragm proteins ZO-1 and nephrin. The merged ZO-1/Actn4 images show overlapping patterns of expression in the wild-type and *Actn4* ^K228E/+^ mice, but clearly distinct expression patterns in the *Actn4* ^K228E/K228E^ mice.

We examined α-actinin-4 localization in a human *ACTN4* ^K228E/+^ kidney biopsy sample (Figure 6B). Although we are cautious in our interpretation, given the availability of only one biopsy sample, there appears to be a more punctate appearance to the α-actinin-4 distribution, consistent with the existence of some α-actinin-4 aggregates in human heterozygotes.

## Discussion

We have previously shown that dominantly inherited point mutations in the α-actinin-4 gene *ACTN4* cause a form of human glomerular disease (Kaplan et al. 2000). We have also shown that mice lacking α-actinin-4 expression develop a severe glomerular lesion (Kos et al. 2003). Here we have further explored the biochemical and cell biologic alterations caused by disease-associated α-actinin-4 mutations.

Human α-actinin-4-associated FSGS is characterized by a dominant pattern of inheritance. Affected individuals typically develop disease in adulthood. Some individuals develop progressive renal failure, others develop moderate proteinuria, while a few show no evidence of kidney dysfunction well into adulthood. This contrasts with other recently elucidated inherited disorders of the podocyte caused by mutations in the slit-diaphragm proteins nephrin and podocin, where disease typically presents in the neonatal period or in childhood and follows a recessive pattern of inheritance (Kestila et al. 1998; Boute et al. 2000). Mice lacking slit-diaphragm proteins CD2AP and Neph-1 similarly present with very early-onset nephrosis (Shih et al. 1998; Donoviel et al. 2001). Furthermore, in contrast to the typically diffuse podocyte foot process effacement observed in kidney biopsies from individuals with these recessive forms of disease, individuals with *ACTN4*-associated FSGS have focal podocyte abnormalities, nonnephrotic levels of proteinuria, and slowly progressive adult-onset disease leading to significant (and often end-stage) renal failure in adulthood.

These phenotypic differences themselves suggest a different mechanism of disease from what is observed with slit-diaphragm protein defects. Our earlier experiments suggested that mutant α-actinin-4 binds actin filaments more strongly than wild-type α-actinin-4 in vitro. However, this may reflect a propensity toward oligomerization rather than increased F-actin binding per se. The finding that the mutant α-actinin-4 forms aggregates with greatly decreased half-life suggests two possible models to explain the human (and mouse) disease. One model would explain the development of podocyte damage as a direct effect of protein aggregation and the toxic effects of such aggregation, as is observed in several degenerative neurologic conditions such as Alzheimer and Parkinson diseases (Horwich 2002). The second model explains the disease as a loss-of-function disease, reflecting the increased rate of mutant α-actinin-4 degradation.

We do not regard these models as mutually exclusive. In fact, we believe it likely that the development of disease may involve both of these mechanisms. It is interesting to note that α-actinin-4-mediated kidney disease bears some similarities to the adult-onset neurodegenerative condition Huntington disease. In Huntington disease, dominant mutations that lead to expanded polyglutamine tracts cause neurodegeneration. The mutant huntingtin protein is misfolded and forms aggregates that are thought to have dominant, toxic effects on neuron function (Bucciantini et al. 2002; Bates 2003). These proteins also play critical and nonredundant roles in the relevant organs: analogous to what we have observed in α-actinin-4-deficient mice, mice with reduced huntingtin expression show abnormal brain development and perinatal lethality (White et al. 1997).

In contrast to humans, mice heterozygous for α-actinin-4 mutations do not have overt disease. We suspect that in humans, over a timespan of several decades, the combination of decreased α-actinin-4 expression and the formation of aggregates ultimately proves toxic. We suggest that the relatively short life of mice compared with that of humans may be the major explanation of this difference. Not all humans carrying disease-associated mutations develop clinical disease (Kaplan et al. 2000). Disease, in both human and murine heterozygotes, likely requires a “second hit,” either in the strict genetic sense of a second mutation in the relevant cell type or a “physiologic hit,” such as elevated blood pressure, renal toxin exposure, or vascular disease, to name three examples. We suspect that renal stresses will uncover deleterious renal phenotypes in heterozygous mice, similar to the disease observed in humans. With aging and gradual accumulation of aggregates in the terminally differentiated podocyte, we believe that humans with *ACTN4* mutations become increasingly susceptible to minor insults. We note also that mice, unlike humans, express α-actinin-1 in podocytes (Kos et al. 2003). This may help stabilize mutant α-actinin-4 or may produce more redundancy, giving the mouse glomerulus greater protection to perturbations in this pathway.

**Figure 7:**
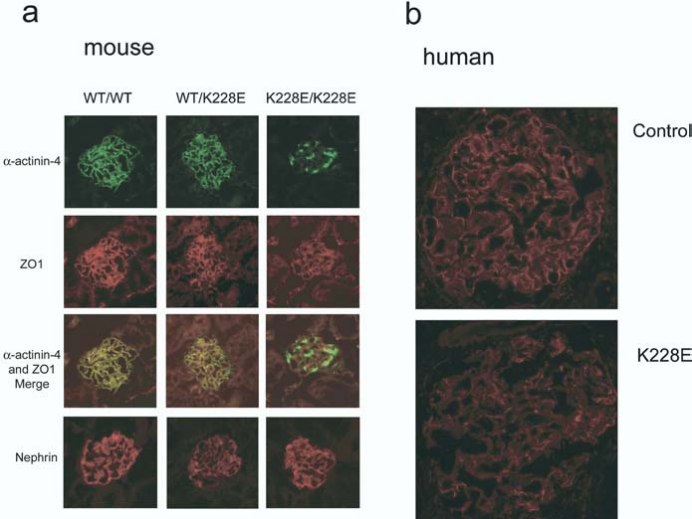
In Situ Protein Localization (A) IF studies of glomerular protein expression in *Actn4* ^+/+^, *Actn*4^K228E/+^ , and *Actn4* ^K228E/K228E^ mice. As indicated, expression of α-actinin-4, ZO-1, and nephrin is shown, as is a merged image of α-actinin-4 and ZO-1 expression. (B) Glomerular expression of α-actinin-4 in normal human kidney and in an individual heterozygous for a K228E mutation.

We note that, as shown in Figure 7, the appearance of α-actinin-4 is more punctate in the kidney from a K228E heterozygous individual than a control, consistent with the existence of aggregates in heterozygous humans. This effect, however, is subtle, and is consistent with the lack of any detectable renal phenotype in several humans who carry disease-associated *ACTN4* mutations (Kaplan et al. 2000). Although the number of *ACTN4* mutations we have found is small, we have not detected any human disease-associated *ACTN4* mutations to date that lead to premature termination, suggesting that simple haploinsufficiency may not by itself cause disease (our unpublished data).

As indicated in Figure 5 and in the text, heterozygous K228E *Actn4* knockin mice have no clear phenotype either by histologic analysis at the light and electron microscopic levels or by analysis of urine protein and serum creatinine. Even at more advanced ages, the *Actn4* ^K228E/+^ mice appear normal. By contrast, we observe clear glomerular phenotypes in both *Actn4* ^–/–^ and *Actn4* ^K228E/K228E^ mice. Our genetic observations are consistent with our biochemical observations. Specifically, we believe that α-actinin-4 mutations lead to a reduction in normal α-actinin-4 activity and to protein aggregation and that glomerular phenotypes reflect both loss of normal α-actinin-4 function and toxic effects of aggregated α-actinin-4. While the disease observed in homozygous *Actn4* ^K228E/K228E^ mice may primarily reflect loss of function resulting from rapid α-actinin-4 degradation, heterozygous humans may show slow development of podocyte damage from the effects of α-actinin-4 aggregation.

Is alteration of α-actinin-4 expression or conformation a cause or a mediator of secondary forms of kidney disease? These seem plausible hypotheses given the data presented here, together with results from other investigators showing alterations in α-actinin-4 levels in association with proteinuria in certain animal models (Shirato et al. 1996; Smoyer et al. 1997). This suggests that physiologic processes that alter the expression, conformation, or both of this (and other) cytoskeletal proteins, either at the protein or the transcript level, might be amenable to interventions that restore normal patterns of protein expression.

## Materials and Methods

### 

#### Cell and cell culture**

Mouse podocytes were kindly provided by Dr. Peter Mundel (Albert Einstein College of Medicine, Bronx, New York, United States) and cultured as described previously (Mundel et al. 1997). In brief, undifferentiated podocytes were cultured in RPMI-1640 (Cellgro, CellGenix, Freiburg, Germany) medium containing 10% fetal calf serum (FCS) and 20 U/ml γ-interferon (γ-IFN) at 33°C and 5% CO_2_. Differentiated podocytes were cultured in the medium containing no γ-IFN at 37°C. Additional conditionally immortalized podocytes from “knockin” litters were generated exactly as described previously (Mundel et al. 1997). Fibroblasts were derived from lungs dissected from newly sacrificed adult mouse littermates of different genotypes. Cells were propagated in culture and immortalized by transfection with an SV-40 large T-antigen expression plasmid using Fugene 6 transfection reagent (Roche, Basel, Switzerland).

#### Protein extraction

Fibroblasts were allowed to grow to confluence, then scraped off the tissue culture plate in the presence of cold phosphate-buffered saline (PBS) and spun at 3,000 rpm at 4°C for 10 min. Lymphocytes were isolated from whole blood with Histopaque-1077 solution (Sigma, St. Louis, Missouri, United States) following the manufacturer's instructions. The pellets were resuspended in ice-cold lysis buffer (150 mM NaCl, 50 mM Tris [pH 8.0], 1% Triton X-100, 1 mM Na-orthovanadate, 4 μM microcystin, and protease inhibitor). We collected the supernatant and estimated the protein concentration using either the Bradford method or equalizing the protein concentration in different lysates by Western blot using β-actin as a standard.

#### Transient transfection and Immunocytochemistry

We mutated a wild-type pBluescript-GFP-ACTN4 clone using a QuickChange kit (Stratagene, La Jolla, California, United States) to create clones harboring each of three disease-associated mutations (K228E, T232I, S235P) (Kaplan et al. 2000). These mutant and wild-type α-actinin-4 clones were transfected into podocytes using Fugene 6 (Roche). Cells 60 h after transfection were fixed in 2% paraformaldehyde and 4% sucrose in PBS for 5 min and then permeabilized in 0.3% Triton for 5 min. Fixed cells were blocked with 2% FCS, 2% BSA, 0.2% fish gelatin in PBS for 60 min and incubated with anti-α-actinin-4 antibody, and rabbit antigen–antibody complexes were visualized with fluorochrome-conjugated secondary antibodies.

#### Sucrose gradient studies

Sucrose gradients of 5%–20% and 10%–40% were made using a buffer containing 0.02 M Tris–HCl (pH 7.5), 0.15M NaCl, 0.1 mM EDTA, and 0.2 mM of DTT and were internally calibrated with BSA, carbonic anhydrase, and catalase. In vitro translated and radiolabeled wild-type and mutant α-actinin-4 (K228E, T232I, S235P) were loaded onto the gradient, centrifuged at 40,000 rpm for 15 h at 4°C, and eluted into 0.2 ml fractions. Eluates were analyzed by SDS-PAGE and autoradiography.

#### Pulse–chase studies**

Immortalized mouse fibroblasts were incubated in methionine-free MEM medium containing 10% FCS (dialyzed overnight using 12K-14K SPECTRA/POR porous membrane in normal saline at 4°C) and 25 mM HEPES for 20 min at 37°C. [^35^S]Methionine was added to a final concentration of 0.1 mCi to pulse the cells. Cells were pulse labeled for 0, 15, 30, 60, 120, 180, and 240 min. To study the degradation of α-actinin-4, cells were pulsed for 3 h and then chased for 0, 3, 6, 12, 20, 24, and 30 h with excess cold methionine. We used anti-α-actinin-4 antibody recognizing the N-terminus to precipitate α-actinin-4 from the cell lysates. Protein A–sepharose beads (Pierce Biotechnology, Rockford, Illinois, United States) were preincubated with the anti-α-actinin-4 antibody for 2 h at 4°C and then incubated with the lysates overnight at 4°C. The beads were washed with lysis buffer and resuspended in SDS-PAGE loading buffer. Samples were resolved on a 10% polyacrylamide gel and visualized by exposure to radiographic film. For lactacystin treatment, cells were first pulsed for 3 h as above, followed by addition of 2.5 μM lactacystin dissolved in DMSO (A. G. Scientific, San Diego, California, United States) or 0.125% DMSO alone with cold methionine.

#### Nuclear DNA injection and imaging

For injection and imaging, cells were cultured on MatTek Corporation (Ashland, Massachusetts, United States) 35 mm coverslip dishes in F12 media (BioFluids, BioSource International, Carmarillo, California, United States) without phenol red and supplemented with 10 mM HEPES and antibiotics. Plasmid DNA at 0.5–2.0 ng/nl was injected in cell nuclei using a Narishige (Lake Forest, California, United States) IM-200 picoliter pressure injection system. OD microcapillary glass pipettes (1.0 mm) were pulled to a fine tip using a Narishige PB-7 needle puller. Cells were maintained at 37°C using a modified Harvard Apparatus (Hopkinton, Massachusetts, United States) microscope incubator mounted on a Nikon (Tokyo, Japan) Diaphot 300 inverted microscope. Images were collected using a Princeton Instruments MicroMax 1300Y cooled CCD camera (Roper Scientific, Tucson, Arizona, United States). Excitation and emission wavelengths were controlled using dichroic and bandbass filters from Omega Optical (Brattleboro, Vermont, United States) and a Sutter Instrument (Novato, California, United States) Lambda 10–2 filter wheel image acquisition. Device control and postacquisition processing were done with Isee Imaging Software (Inovision Corporation Raleigh, North Carolina, United States).

#### Actinin dynamics display

To display the changes in GFP–actinin distribution over time, images collected at 1 min intervals were used sequentially as red, green, and blue channels of a RGB composite image. Areas of signal that did not change have equal representation in each of the channels and generate a white signal in the final image. Areas of signal that did change on a minute-to-minute basis are indicated by either a red, green, or blue hue. For example, a region rich in red would indicate an signal present in the first image, but absent in the two sequential images, indicative of a withdraw or loss of signal in that region.

#### Generation of K228E mutant mice

Previously, we described the development of a mouse model lacking detectable *Actn4* expression (Kos et al. 2003). These mice harbor a germline mutation in exon 8 of *Actn4* encoding a K228E substitution, as well as an intronic *loxP*-flanked neomycin resistance cassette. We bred these mice to transgenic mice with germline expression of *Cre* recombinase (129-TgN(Prm-Cre)58Og; Jackson Laboratory, Bar Harbor, Maine, United States). We verified excision of the neomycin resistance cassette by PCR. Heterozygous mice were crossed to obtain mice homozygous for the K228E substitution. Mice were genotyped for the K228E as described previously (Kos et al. 2003). We used Trizol reagent to extract RNA from kidneys for RT-PCR and for Northern blot analysis.

#### Mouse phenotyping

Freshly harvested kidneys were fixed in Bouin's solution. H & E and PAS staining were performed using standard methodology. Electron microscopy was performed after fixation in Karnovsky's media using standard diagnostic protocols. For the electron micrographs, all of the glomeruli imaged were from as deep into the renal cortex as possible. Indirect IF studies were performed using standard methods (Kos et al. 2003). Urine microalbumin was assessed by a reader blinded to mouse genotype using albumin dipsticks (Albustix, Bayer, Leverkusen, Germany). BUN and creatinine measurements were performed at the clinical chemistry laboratory at Brigham and Women's Hospital.

## Supporting Information

Video S1 Podocytes Following Nuclear Injection of Wild-Type GFP-α-Actinin-4 cDNA (14.8 MB MOV).

Video S2 Podocytes Following Nuclear Injection of K228E GFP-α-Actinin-4 cDNA (11.8 MB MOV).

Video S3 Podocytes Following Nuclear Injection of T232I GFP-α-Actinin-4 cDNA (10.9 MB MOV).

### Accession Numbers

GenBank accession numbers for genes and proteins discussed in this paper are NM_004924 and NP_004915 (human *ACTN4*; also LocusLink ID 81) and NM_021895 and NP_068695 (mouse *Actn4*; also LocusLink ID 60595). OMIM numbers are 603278 (*FSGS-1*) and 604638 (*ACTN4*).

## Abbreviations

BUN: blood urea nitrogen
FCS: fetal calf serum
FSGS: focal segmental glomerulosclerosis
GFP: green fluorescent protein
H & E: hematoxylin and eosin
IF: immunofluorescence
IFN: interferon
PAS: periodic acid–Schiff
PBS: phosphate-buffered saline
